# Simulation and Experimental Study of Ion Concentration Polarization Induced Electroconvective Vortex and Particle Movement

**DOI:** 10.3390/mi12080903

**Published:** 2021-07-29

**Authors:** Junghyo Yoon, Youngkyu Cho, Jaehoon Kim, Hyunho Kim, Kyuhwan Na, Jeong Hoon Lee, Seok Chung

**Affiliations:** 1 School of Mechanical Engineering, Korea University, 145 Anam-ro, Seoungbuk-gu, Seoul 02841, Korea; jhyoon7102@gmail.com (J.Y.); kimhuks@korea.ac.kr (J.K.); khh8518@korea.ac.kr (H.K.); nagoon123@gmail.com (K.N.); 2 Department of IT Convergence, Korea University, 145 Anam-ro, Seoungbuk-gu, Seoul 02841, Korea; ykcho1993@gmail.com; 3 Smart Device Team, Samsung Research, Samsung Electronics Co., Seoul R&D Campus, 34 Seoungchon-gil, Seocho-gu, Seoul 06765, Korea; 4 Absology Co., Ltd., Anyang 14057, Korea; 5 Department of Electrical Engineering, Kwangwoon University, 20 Kwangwoon-ro, Nowon-gu, Seoul 01897, Korea; 6 KU-KIST Graduate School of Converging Science and Technology, Korea University, Seoul 02841, Korea

**Keywords:** ion concentration polarization, electroconvective vortex, manipulation, ion-permselective material

## Abstract

Ion concentration polarization (ICP) has been widely applied in microfluidic systems in pre-concentration, particle separation, and desalination applications. General ICP microfluidic systems have three components (i.e., source, ion-exchange, and buffer), which allow selective ion transport. Recently developed trials to eliminate one of the three components to simplify the system have suffered from decreased performance by the accumulation of unwanted ions. In this paper, we presented a new ICP microfluidic system with only an ion-exchange membrane-coated channel. Numerical investigation on hydrodynamic flow and electric fields with a series of coupled governing equations enabled a strong correlation to experimental investigations on electroconvective vortices and the trajectory of charged particles. This study has significant implications for the development and optimization of ICP microfluidic and electrochemical systems for biomarker concentration and separation to improve sensing reliability and detection limits in analytic chemistry.

## 1. Introduction

**Figure 1:**
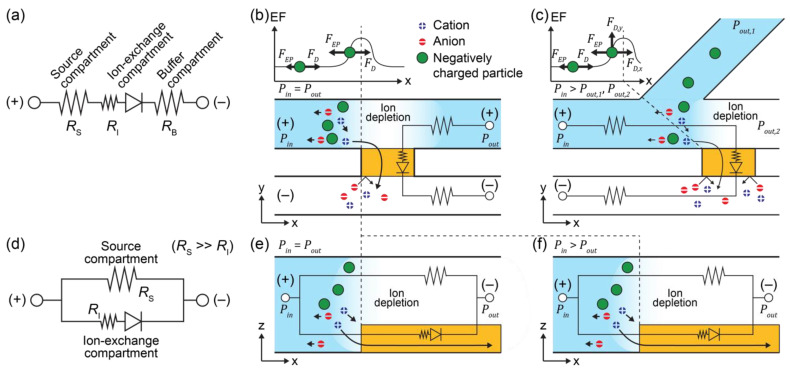
Ion concentration polarization (ICP)-microfluidic system. (**a**) Equivalent circuit of ICP-3C system consisting of source, buffer, and ion-exchange compartments. ICP-3C with an application of (**b**) electric field (EF) (ICP-3C-EF) [8,13] and (**c**) electric and hydrodynamic flow fields (HF) (ICP-3C-EF/HF) [10,14,15]. (**d**) Equivalent circuit of ICP-2C system, consisting of source and ion-exchange compartment. ICP-2C with an application of (**e**) electric field (ICP-2C-EF) [17,18] and (**f**) electric and hydrodynamic flow fields (ICP-2C-EF/HF) [11,12,19]. $R_{S}$, $R_{B}$, and $R_{I}$ indicate resistances of source, buffer, and ion-exchange compartment, respectively.

Ion concentration polarization (ICP) is a fundamental electrokinetic phenomenon resulting from selective ion transport through ion-exchange films, such as cation-exchange membranes (CEM) or anion-exchange membranes (AEM), which allow only cations or anions to pass [1,2,3]. Conventional electromembrane processes (e.g., electrodialysis) follow a typical system configuration in which spacers (i.e., channels) and ion-exchange membranes are alternatively stacked between electrodes to operate under two vertically applied electric and hydrodynamic flow fields [4,5]. After the electric potential is applied across CEM/AEM, the ion-depleted/enriched diffusion layer on the anodic side of the membrane is triggered by selective ion transport accompanying the ICP. A thicker ion depletion layer that develops beyond a diffusion-limited thickness leads to an electroconvective instability. The thicker layer also results in the reduction of system performance owing to the limitation of ion diffusivity and the degradation of membrane selectivity by highly depleted or accumulated ions on the membrane surface [3,6,7]. Recently, microfluidic systems have facilitated a well-defined steady-state electroconvection, owing to the advantages of spatial controllability in microfluidic systems, the application of in micro- and nanofluidic-scale pre-concentration bio-agents [8,9], the separation of charged particles [10,11], and fluidic mixing [12]. ICP microfluidic systems have various configurations according to device compartments and forces applied to control target particles for corresponding applications (Figure 1 and Table 1).

A traditional ICP microfluidic system which comprises three components (ICP-3C) was depicted as an equivalent circuit model (Figure 1a). The first component is a source, and the second is a buffer where an anode and a cathode are generally placed. The third is an ion-exchange channel compartment (e.g., CEM or negatively charged nanochannel junction), which allows the movement of counter-ions (i.e., cations in the case of CEM) between the two compartments [2,8,13,14]. The source and buffer compartments can be described as resistances $R_{S}$ and $R_{B}$, respectively, and the diode represents the ion-exchange compartment with an internal resistance of $R_{I}$ ($R_{I}\ll R_{S}$). Target particles (i.e., ions, deoxyribonucleic acids (DNAs), proteins, or cells) are placed adjacent to the anodic side of the ion-exchange compartment alongside the source compartment. The buffer compartment collects the relocated counter-ion after it passes through the ion-exchange compartment. The ICP-3C system is classified into two applied categories according to external force fields: a single field (electric field (EF) only, ICP-3C-EF, Figure 1b) [8,13] and a coupled field (EF and hydrodynamic flow fields (HF), ICP-3C-EF/HF, Figure 1c) [14,15]. The ICP-3C-EF system develops an ion depletion layer next to the anodic side of the ion-exchange compartment and generates a spatially redistributed electric field (EF) [8,9,16]. The redistributed EF forms an electro-osmotic flow alongside the source compartment and an electroconvective vortex (EV) on the anodic side of the ion-exchange compartment. The equilibrium of electrophoretic and drag forces on particles results in the enrichment or separation of bio-agents to detect concentrated or separated bio-agents in specific areas of the channel [8,13]. The ICP-3C-EF/HF system, in which an additional HF allows continuous control of target particles, interrupts the flow of nano- and micro-sized particles and lets them be collected or detected from the channel for further processing [10,14,15]. In spite of their usability, ICP-3C systems suffer from an accumulation of ionic species (i.e., transported counter-ions and newly generated chemicals by the electrode) in the buffer compartment, causing deterioration of the perm-selectivity of the ion-exchange compartment. Additional washing is therefore required to maintain stable operation.

Instead of ICP-3C systems, a two-compartment configuration (ICP-2C) having only source and ion-exchange channel compartments was suggested (Figure 1d) [12,17,18,19]. The new configuration directly connects the anode and cathode via the source compartment. Ions in the source compartment can freely move toward their corresponding electrodes, and the counter-ions move through the ion-exchange compartment rather than the source compartment, owing to the high conductivity of the ion-exchange compartment toward the counter-ion ($R_{S}\gg R_{I}$). The counter-ion movement creates a higher electric field near the anodic side of the ion-exchange compartment, enabling protein concentration [17,18]. When integrated with additional HF, the ICP-2C-EF/HF system increased throughput to extract the target particle in the preconcentration process [11,12,19]. Compared with the ICP-3C systems which suffer from ion accumulation in the buffer compartment, the ICP-2C system has distinct merits in terms of its continuous discharge of the waste buffer and simple structures [8,17]. However, the ICP-2C system has not been fully optimized with numerical analyses by applying the three coupled governing equations of Nernst–Plank, Navier–Stokes, and Poisson (Table 2). This study develops an axial two-dimensional (2D) symmetry model of the ICP-2C-EF/HF system with coupled governing equations. Numerical predictions of the electroconvective vortex and movement of charged particles were confirmed experimentally under various hydrodynamic and electric fields. Significant implications are expected for the development of ICP microfluidic systems to control biomarkers by improving sensing reliability and detection limits in analytic chemistry.

## 2. Materials and Methods

### 2.1. Experiment Setup

**Figure 2:**
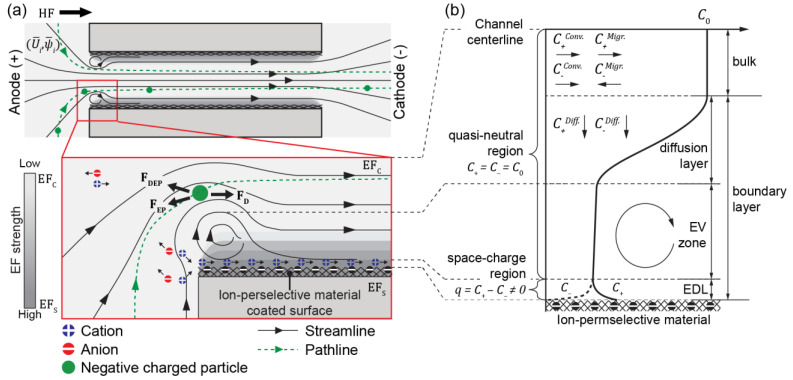
(**a**) Developed electroconvective (EV) and forces experienced by the charged particle in 2C-Par-ICP system. (**b**) Structure of ion concentration (IC) boundary layer at the inlet of ICP-2C-EF/HF.

The experimental data were generated using a microfluidic device for the ICP-2C-EF/HF system, whose configuration, fabrication, and operation have been described and demonstrated in previous works [11,19]. Figure 2a and Figure S1 show the operation and the schematic depiction of the movement of ions (0.1× phosphate-buffered saline) and particles (0.2 mL^−1^, 1-µm diameter; Invitrogen, Carlsbad, CA, USA). The device was designed to contain two reservoirs connected via an ion-exchange compartment, with Nafion-coated (Sigma–Aldrich, St. Louis, MO, USA) ion-concentration channel 500 μm wide and 120 μm high (hydraulic radius ≈ 100 μm). The platinum electrodes were connected to a source meter (Model 2400, Keithley Instruments, OH, USA) to regulate the electric potential at an inter-electrode spacing of 1 cm.

### 2.2. Theoretical Model and Simulation

Details of the simulation settings (e.g., governing equations, simulation models, and simulation methods) are described in the supporting information (Figure S2 and Table S1). Three coupled governing equations of Nernst–Plank, Navier–Stokes, and Poisson were applied to an axial 2D symmetry model of the ICP-2C-EF/HF system. The governing equations were solved numerically on finite elements using COMSOL Multiphysics (v.5.2, COMSOL, Inc., Stockholm, Sweden). For a simplified expression, the cylindrical geometries, ion concentration, electric potential, and flow velocity are normalized as described:

(1) $$\begin{array}{c} \begin{array}{c} \left(\bar{r},\bar{z}\right)=\left(\frac{r}{r_{0}},10\frac{z}{z_{0}}\right)\\ {\bar{C}}_{+}=\frac{C_{+}}{C_{0}},{\bar{C}}_{-}=\frac{C_{-}}{C_{0}}\\ \bar{\psi }=\frac{\psi }{\psi_{0}}\\ \bar{U}=\frac{U\left(u,w\right)}{U_{0}} \end{array}\\ \left({\bar{U}}_{\mathrm{in}},{\bar{\psi }}_{\mathrm{in}}\right)=\left(\frac{U_{\mathrm{in}}}{U_{0}},\frac{\psi_{\mathrm{in}}}{\psi_{0}}\right) \end{array}\},$$

where $r_{0}$ (=100 μm) is the equivalent radius of the ion-permselective coated channel, and $z_{0}$ (=1000 μm) is the length scale of the channel length. $C_{0}$ (=0.1 mM) is the bulk concentration in the electrolyte solution with a diffusivity of 1.6 × 10^−9^ m^2^/s for both cations and anions. $\psi_{0}$(=1 V) is the reference electric potential to achieve an electric field of the same order as the experimental condition (=10–30 V/cm). $U_{0}$ (=0.106 mm/s, *Q*_0_ = 2 µL/min) is the reference average velocity.

## 3. Results

### 3.1. ICP and Electroconvective Vortex in ICP-2C-EF/HF System

**Figure 3:**
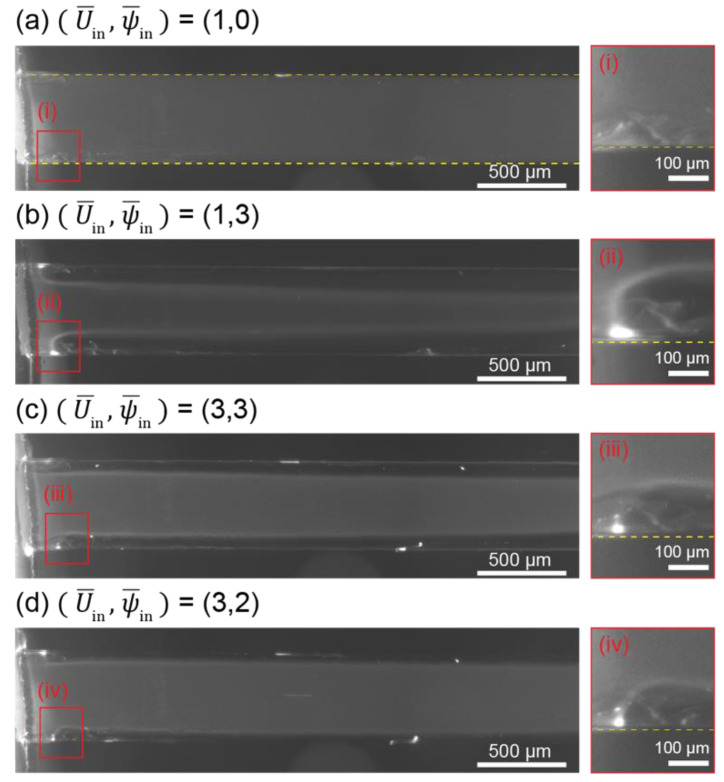
Experimental results of particle movement in ICP-2C-EF/HF with corresponding experiment conditions, $\left({\bar{U}}_{in},{\bar{\psi }}_{in}\right)$ = (**a**) (1,0), (**b**) (1,3), (**c**) (3,3), and (**d**) (3,2). Yellow dotted line indicates the surface of IC-channel. Red rectangles indicate the entrance of IC-channel where depletion layers are developed.

In the ICP-2C-EF/HF, ICP was initiated at the entrance of the ion-exchange compartment, the ion concentration channel (IC channel) forming an electric double layer (EDL), owing to the lower electric resistance at 25 °C of nafion on the surface (3.65 × 10^5^/S) than that of the bulk solution (5.4 × 10^10^/S) [36] (Figure 2a). The electric field ($-\nabla \bar{\psi }$) can be considered as two; one $-\nabla \bar{\psi }$ through the IC-channel ($-\nabla {\bar{\psi }}_{C}$) and the other $-\nabla \bar{\psi }$ near the IC-channel surface ($-\nabla {\bar{\psi }}_{S}$) formed by the space charge described by Poisson’s equation. A higher $\bar{\psi }$ generates electro-osmotic instability because of the limitation of the ionic diffusion transport, which forms a plateau IC profile that leads to an electroconvective vortex (Figure 2b). This profile regulates a local electric field profile (i.e., $-\nabla {\bar{\psi }}_{S}$) that generates an electrophoretic and dielectrophoretic force on a charged particle. The development of an electroconvective vortex is also regulated by the hydrodynamic flow ($\bar{U}$), which works as shear flow, hydrodynamically suppressing the growth of the vortex and reducing ICP by its continuous ion supply. The $\bar{U}$ additionally acts as a drag force on a particle [10,37]. The fully coupled interactions redistribute the flow and electric fields and regulate particle movement. Figure 3 shows experimental streamlines as a function of ${\bar{U}}_{in}$ and ${\bar{\psi }}_{in}$ ($\left({\bar{U}}_{in},{\bar{\psi }}_{in}\right)$). A circular depletion layer (i.e., electroconvective vortex) is observed at the entrance of the IC channel. Lower ${\bar{U}}_{in}$ ((1,3) Figure 3b) facilitates the growth of large-sized electroconvective vortices, while a higher ${\bar{U}}_{in}$ ((3,3) Figure 3c) suppresses growth. Compared to the conventional electromembrane process in which electroconvective vortices are sequentially developed along the channel [38], in the ICP-2C-EF/HF, particles follow a stabilized path after experiencing a dramatic detour at the entrance by a spatially controlled electroconvective vortex.

### 3.2. Effect of Flow and Electric Fields on ICP

**Figure 4:**
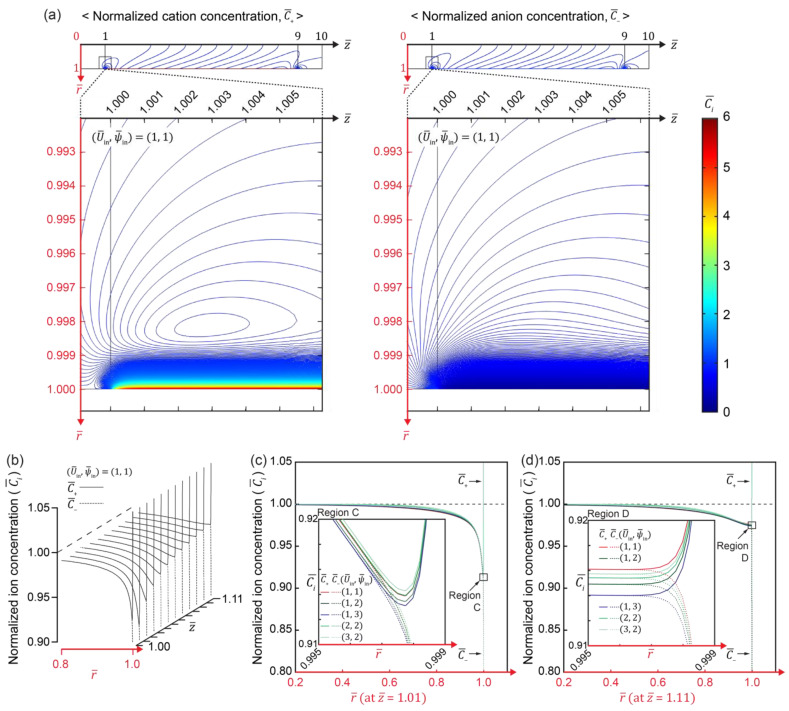
(**a**) Representative contour maps depicting the spatial distribution of IC and (**b**) profiles of IC near IC-channel entrance under $\left({\bar{U}}_{\mathrm{in}},{\bar{\psi }}_{\mathrm{in}}\right)=\left(1,1\right)$. (**c**) Change in IC profile at IC-channel entrance (z = 1.01) and (**d**) at stabilized point (z = 1.11) along the r-axis as a function of ${\bar{U}}_{\mathrm{in}}$ and ${\bar{\psi }}_{\mathrm{in}}$.

Figure 4 shows IC distribution in the IC channel, polarized along the IC channel surface (Figure 4a,b). The typical EDL and diffusion layer ($\bar{z}=1.01,$Figure 4b,c) could be noticed at the tight entrance of the IC channel ($\bar{z}=1.01$). The IC gradient then gradually decreased and stabilized, forming a flattened concentration profile ($\bar{z}=1.11$, Figure 4b,d) similar to that where electroconvective vortices were observed (i.e., EV zone in Figure 1b) [38]. Two distinct ionic distributions of circular and parabolic profiles (Figure 4a) were found, similar to the velocity profiles local at the IC channel entrance and global along the channel. A circular profile of ions similar to the velocity profile developed by the electroconvective vortex at the channel entrance will be discussed later. A parabolic profile of ions is observed along the channel where the Hagen–Poiseuille flow was developed. The profile similarity indicates that the ionic distribution and flow velocity were coupled. Figure 4c,d shows the changes in the IC profile at the entrance ($\bar{z}=1.01$) and stabilized point ($\bar{z}=1.11$) along the $\bar{r}$-axis, respectively, as a function of flow velocity (${\bar{U}}_{\mathrm{in}}$) and electric potential (${\bar{\psi }}_{\mathrm{in}}$). Under a fixed ${\bar{U}}_{\mathrm{in}}$, the increase in ${\bar{\psi }}_{\mathrm{in}}$ led to a thicker depletion layer with a steeper concentration gradient, because more ions migrated through the IC-channel surface as ${\bar{\psi }}_{\mathrm{in}}$ increased. Along the same principle, the increase in ${\bar{U}}_{\mathrm{in}}$ increased the mass flux through the channel, resulting in a thinner depletion layer with a lower concentration gradient.

### 3.3. Electric and Flow Field in the IC-Channel

**Figure 5:**
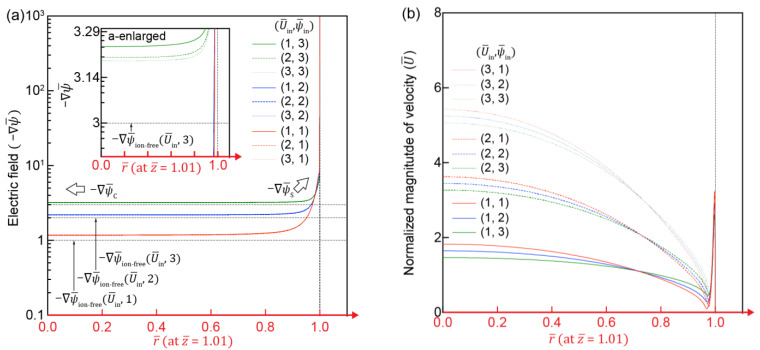
(**a**) Profile of electric field ($-\nabla \bar{\psi }$) along the $\bar{r}$-axis at the IC-channel entrance ($\bar{z}$ = 1.01). Black dotted lines indicate electric fields ($-\nabla {\bar{\psi }}_{\mathrm{in}}$) solely generated by electric potential without the contribution of IC. (**b**) Normalized flow velocity along the $\bar{r}$-axis at the IC-channel entrance ($\bar{z}$ = 1.01).

Figure 5 shows a normalized electric field ($-\nabla \bar{\psi }$) and normalized flow velocity ($\bar{U}$) along the $\bar{r}$-axis at the entrance of the IC channel ($\bar{z}$ = 1.01). $-\nabla \bar{\psi }$ is maximized at the IC channel surface ($-\nabla {\bar{\psi }}_{S}$) due to the field-focusing effect [39] and decreases sharply towards the center of the IC channel ($-\nabla {\bar{\psi }}_{C}$). Notably, $-\nabla {\bar{\psi }}_{C}$ is slightly higher than the electric field formed only from ion-free electric potential ($-\nabla {\bar{\psi }}_{\mathrm{ion}-\mathrm{free}}$), because $-\nabla {\bar{\psi }}_{C}$ is determined by both the application of electric potential (${\bar{\psi }}_{\mathrm{in}}$) and the positive net charge. A higher ${\bar{U}}_{in}$ leads $-\nabla {\bar{\psi }}_{C}$ to approach $-\nabla {\bar{\psi }}_{\mathrm{ion}-\mathrm{free}}$ (Figure 5a-enlarged) due to the reduced electric field by a higher convective flow (i.e., higher mass flux). Figure 5b presents velocities of developing flow near the entrance region. As simulated, flow field in the IC channel was determined by the hydrodynamic and electro-osmotic flows. $\bar{U}$ near the surface of the IC channel is dominated by $-\nabla \psi_{S}$. A higher $-\nabla {\bar{\psi }}_{S}$ leads to high electro-osmotic instability near the channel surface and the development of an electroconvective vortex, which will be discussed in the next section. $\bar{U}$ of the central region of the IC channel (near $\bar{r}=0$) is regulated by $-\nabla \psi_{C}$, and $\bar{U}$ has a relatively flat profile under a higher $-\nabla {\bar{\psi }}_{\mathrm{in}}$ at ${\bar{U}}_{in}=1$, which is known to be the profile of electro-osmotic flow.

### 3.4. Development of Electroconvective Vortex

**Figure 6:**
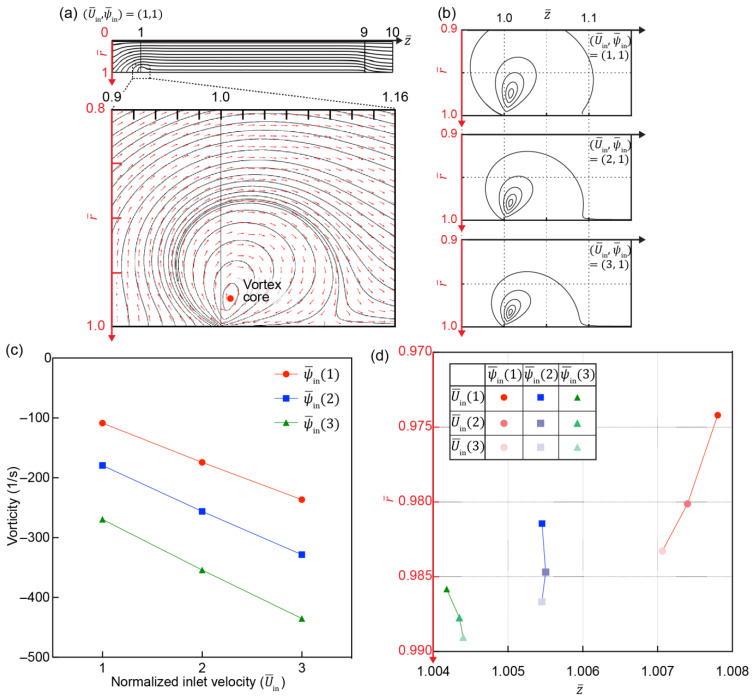
Development of velocity field in the IC-channel. (**a**) Streamlines (black solid-line) and vector of the flow field (red arrows) indicate the developed flow under $\left({\bar{U}}_{in},{\bar{\psi }}_{in}\right)=\left(1,1\right)$ in the IC-channel (streamlines and vector of flow field are shown by the uniform density and the normalized length function of COMSOL, respectively). (**b**) Streamlines of electroconvective vortex. Each is visualized with five streamlines shown by uniform density function of COMSOL. (**c**) Vorticity of electroconvective vortex core and (**d**) its coordinate as a function of ${\bar{U}}_{in}$ and ${\bar{\psi }}_{in}$. Negative sign of vorticity indicates that vortices have a clockwise spin.

The formation of an electroconvective vortex was simulated according to various ${\bar{U}}_{in}$ and ${\bar{\psi }}_{in}$ conditions (Figure 6). The vortex mainly develops near the surface of the IC channel entrance where ICP is significantly developed (Figure 6a). The streamline was plotted to visualize the electroconvective vortex as a function of ${\bar{U}}_{in}$ (Figure 6b), the deformed elliptical shape deflected to the cathode. As ${\bar{U}}_{in}$ increases, the elliptical shape tends to be inclined toward the cathode and decreased in size, suppressed by higher ${\bar{U}}_{in}$. The electroconvective vortex can also be regarded as a forced vortex having a maximum vorticity in the vortex core. Its vorticity and coordinates are shown in Figure 6c,d. The increase in both ${\bar{U}}_{in}$ and ${\bar{\psi }}_{in}$ leads to stronger vorticity and the early onset of the vortex, moving the position of the vortex core to the channel surface and to the IC channel entrance. ${\bar{U}}_{in}$ not only suppresses the growth of the electroconvective vortex but also accelerates its vorticity with high shear stress. A higher ${\bar{\psi }}_{in}$ results in a higher electro-osmotic instability and increases the early onset of the vortex.

### 3.5. Movement of Charged Particles

**Figure 7:**
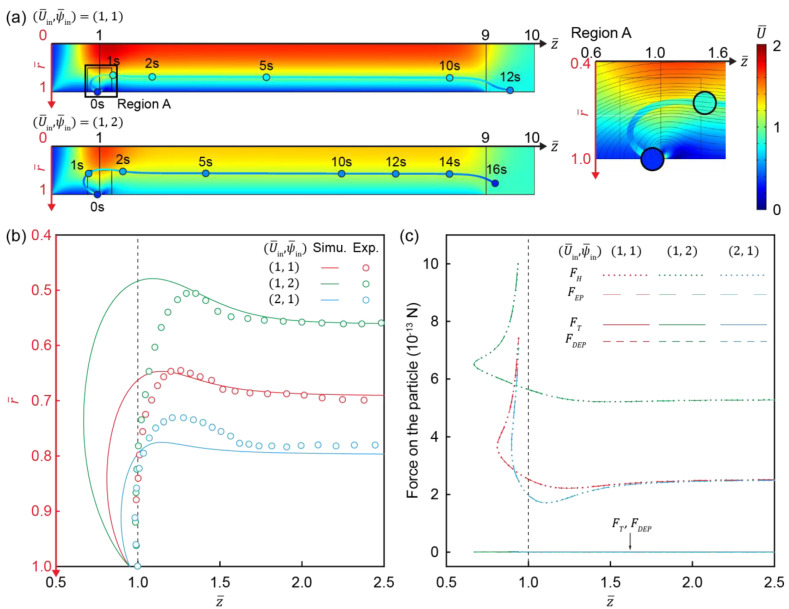
Motion of particle in hydrodynamic flow and electric fields. (**a**) Change in velocity of the flow field and charged particle. Surface color indicates the magnitude of flow-field velocity. Position and color of sphere indicate where particle travels for corresponding time and magnitude of particle velocity, respectively. Path and color of line indicate trajectory and velocity throughout its entire course. The particle trajectory simulation started at (1, 0.95) to avoid a particle trapping in the vortex, because particles started at (1,1) are trapped in the electroconvective vortex (data not shown). (**b**) Simulation and experimental results of the charged particle trajectory and (**c**) forces experienced by the particle as a function of $\left({\bar{U}}_{in},{\bar{\psi }}_{in}\right)$.

The motion of charged particles in flow and electric fields is generally regulated by hydrodynamic drag ($F_{D}$), electrophoretic force ($F_{EP}$), and dielectrophoretic force ($F_{DEP}$) (see supporting information for details) [10,11]. To understand how these forces manipulate the motion of charged particles, a simulation was performed to track their trajectory and applied forces (Figure 7). Flows formed a flattened profile along the channel under a higher ${\bar{\psi }}_{in}$, due to the electro-osmotic flow (color of the surface in Figure 7a). The motion of the particle was mainly regulated by the electric field in the channel ($-\nabla {\bar{\psi }}_{C}$) rather than that near the channel surface ($-\nabla {\bar{\psi }}_{S}$) at its entrance (Region A in Figure 7a). $\nabla \psi_{S}$ is much higher than $\nabla {\bar{\psi }}_{C}$, because $F_{EP}$ generated by $-\nabla {\bar{\psi }}_{C}$ near the channel entrance delays the particle movement toward the channel to form the larger circular trajectory. It placed a particle beyond the influence of $-\nabla {\bar{\psi }}_{S}$ and $\nabla \bar{\psi }\nabla$ (i.e., dielectrophoretic force). A higher ${\bar{\psi }}_{in}$ (i.e., higher $F_{EP}$) slows particle movement toward the channel entrance ($\bar{z}=1$), leading to a shift in the particle trajectory toward the center of the IC channel ($\bar{r}=0$). After particles formed a circular trajectory, they were pushed toward the IC channel surface until the particle trajectory was parallel to the IC channel (Figure 7a,b), where particles experienced no net force (Figure 7c).

## 4. Conclusions

In conclusion, we performed numerical simulations on ICP phenomena in the ICP-2C-EF/HF device under the application of coupled hydrodynamic flow and electric fields. Particle trajectories are stimulated by the interaction of redistributed hydrodynamic flow and electric fields and negatively charged particles with one micrometer of diameter, representing bacteria. The simulated charged particle trajectory under ICP by the charged IC channel surface was validated with experimental results. The generation of an electroconvective vortex at the entrance could be analyzed by its position and velocity, enabling precise tracking of charged particle trajectory under hydrodynamic and electric force fields. The numerical analysis is not only tightly matched with experimental results but also able to provide physical insight into ICP phenomena under parallel force fields to simulate experimental conditions for various applications, such as biomarker concentration and separation to improve sensing reliability and detection limits in analytic chemistry. However, the change in the particle movement according to various particle properties, such as size, charge, and density, within the same force field should be further studied.

## Figures and Tables

**Table 1:** Summary of current ICP-based technologies in the microfluidic platform.

Type	Force Field	Ion-Exchange Compartment	Application	Sample	Refs.
ICP-3C	EF	Nano-channel	ICP layer analysis	Fluorescence	[20]
		Wrinkled nano-channel	Concentration	β-phycoerythrin	[9]
		Patterned nafion	Concentration	β-phycoerythrin	[8]
		Patterned nafion	Concentration	Enzyme	[21]
		Cracked nano-channnel	Concentration	Bovine serum albumin	[22]
		Patterned nafion	Concentration	Albumin	[23]
		Patterned nafion	Concentration	DNA	[13]
	EF/HF	Patterned nafion	Separation	Red blood cell, *E. coli*	[14]
		Patterned nafion	Separation	Micro- and nano-sized particle	[10]
		Patterned nafion	Desalination, separation	Seawater, white blood cell	[15]
		Bipolar electrode	Desalination	Seawater	[24]
		Patterned nafion	Desalination	Salt solution	[25]
		Nafion nanojunction	ICP layer analysis	Potassium chloride	[26]
		Patterned nafion	Concentration	Fluorescence	[27]
		Nafion membrane	Concentration	Hemagglutinin	[28]
ICP-2C	EF	Patterned nafion	Concentration	C-reactive protein	[17]
		Patterned nafion	Concentration	Bacteria	[18]
		Nafion membrane	Concentration	Fluorescence	[29]
	EF/HF	Nano-channel	Flow mixing	Fluorescence	[12]
		Patterned nafion	Concentration	Hemagglutinin	[19]
		Patterned nafion	Focusing	Micro- and nano-sized particle	[11]

EP and HF indicate electric potential and hydrodynamic flow, respectively.

**Table 2:** Summary of current numerical study of ICP-microfluidic system.

Type	Force Field	Boundary Condition for Ion-Exchange Compartment	Ref.
ICP-3C	EF	Fixed volumetric charge	[30]
		Fixed volumetric charge	[31]
		Fixed surface charge and electric potential	[32,33]
	EF/HF	Fixed surface charge	[22]
		Fixed volumetric charge	[34]
		Fixed surface charge and fixed electric potential	[35]
ICP-2C	EF	Fixed volumetric charge	[31]
	EF/HF	Fixed surface charge	This work

All studies applied a two-dimensional (2D) model. The volumetric and surface boundary conditions are constraints for area and line in the 2D model.

## Supplementary Materials

The following are available online at https://www.mdpi.com/article/10.3390/mi12080903/s1, Figure S1: The schematic illustration of the experimental set-up of ICP-2C-EF/HF system. To avoid a fluctuation of a generated bubble by electrolysis, flow generated by hydrostatic pressure difference is implemented with two open reservoirs. Figure S2: Schematic illustration of an ion-permselective material coated channel model and boundary conditions for numerical simulation. Table S1: Parameters used for simulation.
