# Supplementary material for: Simulation and Experimental Study of Ion Concentration Polarization Induced Electroconvective Vortex and Particle Movement
Source: Micromachines (Basel). 2021 Jul 29;12(8):903. doi: 10.3390/mi12080903 (PMC8401646; doi:10.3390/mi12080903)
Supplement: Supplementary file 1 [file micromachines-12-00903-s001.zip › micromachines-1298985-supplementary.pdf]

Supporting information

Simulation and experimental study of Ion Concentration Polarization induced Electroconvective Vortex and Particle Movement

Junghyo Yoon[1], Youngkyu Cho[2], Jaehoon Kim[1], Hyunho Kim[1,3], Kyuhwan Na[1,4], Jeong Hoon Lee[5*] and Seok Chung[1,4,6*]

[1]*School of Mechanical Engineering, Korea University, 145 Anam-ro, Seoungbuk-gu, Seoul 02841, Republic of Korea*

[2]*Department of IT convergence, Korea University, 145 Anam-ro, Seoungbuk-gu, Seoul 02841, Republic of Korea*

[3]*Center for Systems Biology, Massachusetts General Hospital, Boston, MA 02114, U.S.A.*

[4]*Absology Co.Ltd., Anyang 14057, Republic of Korea*

[5]*Department of Electrical Engineering, Kwangwoon University, 20 Kwangwoon-ro, Nowon-gu, Seoul 01897, Republic of Korea*

[6]*KU-KIST Graduate School of Converging Science and Technology, Korea University, Seoul 02841, Republic of Korea*

[*] Correspondence to: Jeong Hoon Lee (E-mail: jhlee@kw.ac.kr),
Seok Chung (E-mail: sidchung@korea.ac.kr)

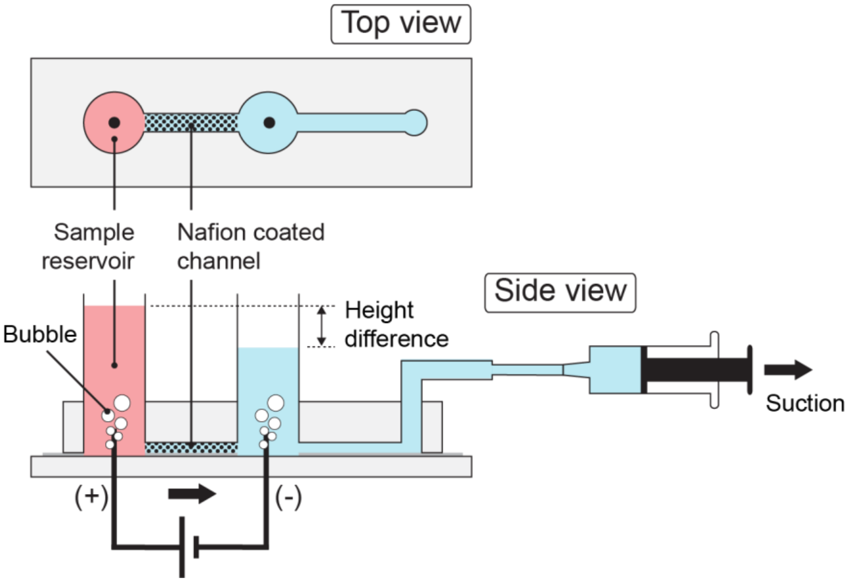

Figure S1 The schematic illustration of the experimental set-up of ICP-2C-EF/HF system. To avoid a fluctuation of a generated bubble by electrolysis, flow generated by hydrostatic pressure difference is implemented with two open reservoirs.

1 Simulation set-up

1.1 Governing equations

Three governing equations, the Nernst-Plank, the Navier-Stokes, and the Poisson equations, are independently solved to obtain simulation results of the ion concentration polarization (ICP) phenomena and three force equations, hydrodynamic, electrophoretic, and dielectrophoretic forces, are applied to determine the motion of charged particles in the ion-permselective material coated channel. The ICP phenomena is fully coupled with ionic concentration of ith species (C_i), vector of fluid velocity ($\mathbf{U_f}$) and electric potential (ψ). The simulation is performed by solving the set of governing equations directly. Firstly, the Nernst-Plank equation (Eq. S1) is applied to ion transport of different species of ions. Secondly, the Navier-Stokes (Eq. S2) and fluidic continuity equation (Eq. S3) is in charge of fluid motion in the channel. Thirdly, the Poisson equation (Eq. S4) is for the electric potential field variation of the ion concentrations. All equations are shown as follows,

$$\frac{\partial C_i}{\partial t} = \nabla \cdot \left(D_i \nabla C_i + z_i D_i \frac{F}{RT} C_i \nabla \psi \right) - \mathrm{U}_f \cdot \nabla C_i \qquad \text{(Eq. S1)}$$

$$\rho \frac{\partial \mathrm{U}_f}{\partial t} + \rho \mathrm{U}_\mathrm{f} \cdot \nabla \mathrm{U}_f = -\nabla p + \mu \nabla^2 \mathrm{U}_f - F \sum_i z_i C_i \nabla \psi \qquad \text{(Eq. S2)}$$

$$\nabla \cdot \mathrm{U}_\mathrm{f} = 0 \qquad \text{(Eq. S3)}$$

$$\nabla^2 \psi = -\frac{F}{\varepsilon} \sum_i z_i C_i \qquad \text{(Eq. S4)}$$

where C_i, D_i and z_i are the concentration, diffusion coefficient and ionic valence of ith ion species in an electrolyte solution, respectively. F is the Faraday constant, R is the ideal gas constant and T is absolute temperature. ρ, μ and ε are the density, viscosity and permittivity of medium.

The motion of charged particle is simulated using the resultant forces ($\mathrm{F_T}$) experienced by a particle throughout the channel (Eq. S5). The forces are hydrodynamic (Eq. S6), electrophoretic (Eq. S7) and dielectrophoretic force (Eq. S8), as described by the following equations :

$$\sum \mathrm{F_T} = \mathrm{F_H} + \mathrm{F_{EP}} + \mathrm{F_{DEP}} \qquad \text{(Eq. S5)}$$

$$\mathrm{F_H} = -6\pi\mu d_p \left(\mathrm{U_p} - \mathrm{U}_f \right) \qquad \text{(Eq. S6)}$$

$$\mathrm{F_{EP}} = \gamma 6\pi \zeta_p \varepsilon d_p \nabla \psi \qquad \text{(Eq. S7)}$$

$$\mathrm{F_{DEP}} = -2\pi\varepsilon d_p^3 (\nabla\psi \cdot \nabla) \nabla\psi \qquad \text{(Eq. S8)}$$

where d_p and ζ_p are the diameter and zeta potential of particle with $\mathrm{U_p}$ being the velocity vector of charged particle. $\gamma = \zeta_n / \zeta_p$ denote the ratio of the zeta potential of the particle to that of the channel wall.

1.2 Simulation method

The governing equations (Eq. S1-S8) are solved numerically using COMSOL Multiphysics (ver. 5.2), which is based on a finite element method. Whereas the Eq. S1-S4 for simulating ICP phenomena used

stationary solver, generating equations without time derivatives, the Eq.S5-S8 for simulating the motion of charged particle used time dependent solver, generating equations for transient (time-dependent) simulation.

1.3 Simulation model

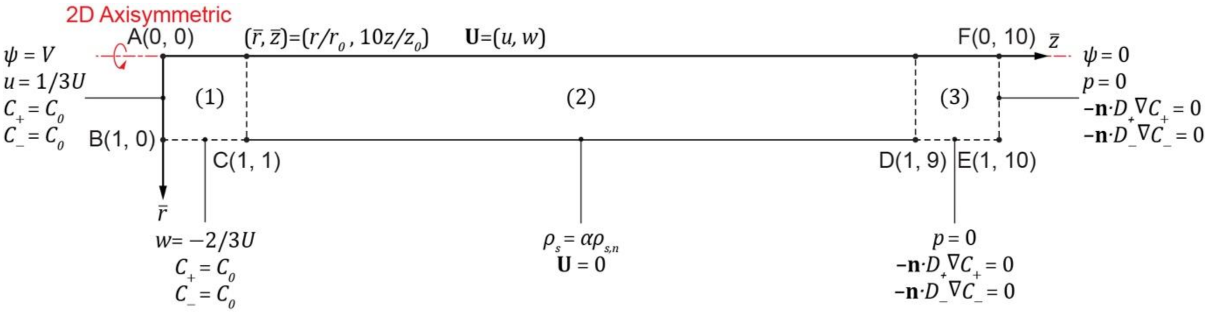

Figure S2 Schematic illustration of an ion-permselective material coated channel model and boundary conditions for numerical simulation.

To reduce the size of a finite element model, we applied axially symmetric geometries (the axis of rotation is $\bar{z}$-axis, 2D Axisymmetric model in COMSOL) under an assumption that the ion-permselective material is homogeneously coated on the channel surface. The model was designed to contain two reservoirs ((1) and (3) in **Figure S2**) connected via ion-permselective material coated channel ((2) in **Figure S2**).

Boundary conditions are set according to the governing equation as given in **Figure S2**. The convective flow is applied between the inlet ($\overline{\text{AB}}$ and $\overline{\text{BC}}$) and the outlet ($\overline{\text{DE}}$ and $\overline{\text{EF}}$). The no-slip boundary condition for fluidic flow is set at the channel surface. The constant ion concentration of electrolyte ($C_{\pm} = C_0$) is enforced at the inlet and the convection dominated transport is set across the outlet ($-\mathbf{n} \cdot D_i \mathbf{\nabla} C_i = 0$). The electric potential (ψ) and ground are applied at $\overline{\text{AB}}$ and $\overline{\text{EF}}$, respectively. Allowing counter-ions to accumulate and pass through at the channel surface, the permselective materials coated channel ($\overline{\text{CD}}$) is assumed to have a high negative surface charge ($\rho_{S,n}$) with a correction factor, α. Because the simulation

was not converged with the direct application of the value in literature even nano-sized mesh was applied to perform numerical analysis.

Table S1 Parameters used for simulation

Symbol	Description	Value
T	Absolute temperature	293.15 [K]
C_0	Ion concentration in electrolyte solution	100 [μM]
D_+	Diffusivity of cations	$1.6\ \times 10^{-9}$ [m^2/s]
D_-	Diffusivity of anions	$1.6\ \times 10^{-9}$ [m^2/s]
ψ_0	Reference electric potential	1 [V]
U_0	Reference average velocity	2 [ul/min]
ζ_{1000}	Zeta potential for the charged particle with 1000 nm of diameter	−4.52 [mV]
r_0	Radius of ion-permselective coated channel	100 [μm]
z_0	Length of ion-permselectvie coated channel	1000 [μm]
α	correction factor	0.02
$\rho_{S,n}$	Surface charge density	$-0.6\ [C/m^2]$ [1]

2 Supplementary Reference

1 T. Colinart, S. Didierjean, O. Lottin, G. Maranzana and C. Moyne, *Journal of The Electrochemical Society*, 2008, **155**, B244.
